# Reinfection of Dogs with Fish-Borne Zoonotic Trematodes in Northern Vietnam following a Single Treatment with Praziquantel

**DOI:** 10.1371/journal.pntd.0002625

**Published:** 2014-01-02

**Authors:** Sofie Nissen, Lan Anh Thi Nguyen, Stig Milan Thamsborg, Anders Dalsgaard, Maria Vang Johansen

**Affiliations:** 1 Department of Veterinary Disease Biology, Faculty of Health and Medical Sciences, University of Copenhagen, Frederiksberg, Denmark; 2 National Institute for Veterinary Research, Hanoi, Vietnam; Universidad Peruana Cayetano Heredia, Peru

## Abstract

**Background:**

Fish-borne zoonotic trematodes (FZT) infections including liver- and minute intestinal flukes are common in Southeast Asia in both humans and domestic animals eating raw fish and since 2010, the liver flukes are recognised as neglected tropical diseases by WHO. Mass drug treatment with praziquantel is advised for humans, but no recommendations for control of the FZT in the reservoir hosts exist.

**Methodology/Principal Findings:**

A study was conducted to assess the ability of praziquantel treatment for control of FZT in farm dogs in an endemic area in Northern Vietnam. Initially, 101 dogs from 73 households were examined for small trematode eggs in their faeces. Forty seven copro-positive dogs were included in the study. Thirty eight dogs received treatment with a single dose of 40 mg/kg of praziquantel. A group of nine dogs were left untreated. Coprological examination for small trematode eggs was performed on day 0, 3, 10, 30, 60, 90 and 120 post treatment. Farmers were questioned about dog keeping practises. All dogs were copro-negative for small trematode eggs on both day 3 and 10 post treatment. From day 30 onwards previously negative dogs became positive again. The reinfection rates were 26.3% (day 30), 45.5% (day 60), 53.1% (day 90), 61.3% (day 120).The nine untreated dogs remained positive throughout the study period. There was no difference in the intensity of infection at day 0 and 120 neither in the treated or untreated group.

**Conclusion:**

Dogs had easy access to raw fish and did not receive treatment against flukes by their owner. More than 50% of the dogs were reinfected 3 months post treatment. We do not recommend controlling FZT infections in dogs by anthelmintic treatment alone since reinfection occurs fast under the existing farm management systems.

## Author Summary

Fish-borne zoonotic trematodes (FZT) including liver and small intestinal flukes in humans and domestic animals are a growing problem in Southeast Asia. WHO has recently recognized the problem, listed the liver flukes as neglected tropical diseases and recommends mass drug treatment of humans to overcome the diseases. Domestic animals including dogs, cats and pigs contribute to transmission and the infection must be controlled in these animals if the transmission cycle shall be broken. We studied the effect of a single praziquantel treatment in a group of natural infected dogs in an FZT endemic area in Northern Vietnam. The dogs were followed over 4 months after treatment and management practices were described. We found that dogs had easy access to raw fish and did not receive treatment against flukes by their owners. More than fifty per cent of the dogs became reinfected with FZT within 3 months after treatment. We conclude that repeated mass treatments are not applicable and cannot stand alone for control of FZT in dogs. The reinfection pattern in humans treated for FZT needs to be investigated to monitor effect of mass drug treatments and avoid development of drug resistance.

## Introduction

The fish-borne zoonotic trematodes (FZT) in South-east Asia comprise of the liver flukes *Clonorchis sinensis* and *Opisthorchis vivirrini* and a large group of minute intestinal flukes (MIF) with more than 35 species mainly belonging to the family Heterophyidae [1]. Humans and fish-eating animals acquire these zoonotic infections by consumption of raw or undercooked fish. It is estimated that 40 to 50 million people are currently infected with one or several species of FZT [2]. The number of infected animals remains unknown. Mixed infections with MIF and liver flukes are often found both in Thailand, Laos and Vietnam [3]–[5]. However, as the eggs of MIF and liver flukes are indistinguishable by light microscopy, studies based on coprological examinations should not report prevalence of a particular species [6]. E.g. a study performed in Northern Vietnam claimed a prevalence of *C. sinensis* of 26% based on faecal examination [7]. However, examinations in fish in the same area showed a prevalence of only 1.5% for *C. sinensis* and 55.6% for the MIF, *Haplorchis pumilio* [8]. Furthermore, several other studies performed in Northern Vietnam showed that MIF are not only prevalent in humans [4], but also in reservoir hosts [9], [10]: Dogs and cats hosted a range of 12 different FZT species, with prevalence of 35–70% in the Nam Dinh and Nghe An provinces. *C. sinensis* was also found but only in very few individuals. Moreover, these studies documented the importance of the reservoir host, especially dogs, in maintaining transmission of the flukes by contributing substantially to contamination of the environment with eggs [9], [10].

Besides preventive chemotherapy in humans, a part of WHO's strategy to overcome neglected tropical diseases is to focus on veterinary public health [11]. Indeed, given the importance of the reservoir hosts in contamination of the environment, FZT cannot be controlled by treatment of humans alone. Therefore, this study was conducted to provide evidence-based information for recommendations for treatment and control of FZT in domestic animals. For humans the recommended dose is 3×25 mg praziquantel per kg body weight, or a single dose of 40 mg/kg for mass drug treatment [12]. Since a single dose would be the most applicable treatment for free roaming farm dogs it was chosen in the present study. Based on the following knowledge, we suspected that reinfection with FZT would occur fast when dogs were constantly exposed to a diet including raw, infected fish.

The pre-patent period for *C. sinensis* is approximately 30 days [13] whereas it is just 9 days for the prevalent MIF, *H. pumilio* [14]. A previous field trial with praziquantel also gave indications of dogs becoming copro-positive for small trematode eggs 30 days after treatment [15].

The specific objective was to evaluate the ability of a single praziquantel treatment to control FZT infection in farm dogs in Northern Vietnam. We determined the effectiveness of drug treatment on day 3 and 10 post treatment, the time until reinfection occurred as well as the intensity of infections before and after treatment. Fluctuations in egg excretion in a group of untreated dogs were also monitored and information on the practice of keeping dogs on farms was obtained through a questionnaire to describe the dog's exposure to raw fish.

## Materials and Methods

### Study Population and Design

The study was carried out from July–December 2011 in Nghia Lac commune in the Nam Dinh province in Northern Vietnam, an area endemic for FZT, where aquaculture is widespread. Initially, faecal samples from 101 randomly selected dogs from 73 farming households were examined for presence of FZT. Dogs were regarded positive for FZT when finding ‘small trematode eggs’, a term commonly used for FZT egg being shorter than 50 µm [4], [16], in the faeces. The study was designed as a randomised intervention study. Forty-seven dogs yielding faecal samples positive for small trematode eggs were chosen by convenience and assorted into either treated or untreated groups. The treated group consisted of 27 young (<1 year) and 11 older dogs (≥1 year), whereas the untreated group consisted of 4 young and 5 older dogs, respectively. Thirty-eight dogs were treated and nine dogs were kept as controls. Dogs were weighed using a scale and treated with praziquantel tablets (Distocide, ShinPoong Pharma Co., Ltd., Seoul, South Korea) at a single dose of 40 mg/kg body weight at day 0 and faecal samples were examined at day 0, 3, 10, 30, 60, 90 and 120 post treatment (untreated dogs were not examined on day 3 and 10). Dog in the treated group were regarded as cured, when no intact eggs were found in their faecal samples on day 3 and 10 post infection and were regarded as reinfected, when intact small trematode eggs were found in their faeces. Dogs younger than 2 months of age, pregnant bitches, dogs showing clinical signs of disease and vicious dogs were excluded from the study.

### Ethics Statement

The animal protocol followed the EU-guidelines for animal experiments [17]. An ethical review of the study was conducted by the project management of FIBOZOPA project in Vietnam, which provided the funds and by the National Institute of Veterinary Research. These two institutions approved the animal protocol, since no official governmental animal ethics committee exist in Vietnam. Informed, oral consent was obtained from the farmers. Dog were treated at the end of the study.

### Dog Keeping Practices

A member of each household visited for the initial screening for FZT-positive dogs was interviewed (in majority of households the person feeding the dogs) to obtain information about the household's general conditions and practices of keeping dogs, including the feeding of raw fish. Information included presence of fish ponds in household or neighbouring household; total number of dogs in the household; roaming behaviour of dogs; and feeding practices, including whether raw fish or leftover meals with fish were fed to dogs. Farmers were also asked about anthelmintic treatment of their dogs during the last month, how often treatment was performed and finally the type of drug used. Information about the 47 dogs in the treatment study included: sex, age, breed, and body condition score ranging from 1 being gaunt, 3 being ideal and 5 being obese [18] was gathered by trained personal. The body weight was measured at the day of treatment (day 0) using a scale and the dogs were assigned an ID number.

### Parasitological Examinations

Faecal samples were collected rectally from the dogs and analysed for FZT eggs by a modification of the method by Willingham et al. [19]. Five gram of faeces was dissolved to make a 100 ml 0.9% saline solution. The sample was washed with saline through three sieves: 400, 100 and 45 µm mesh size. Between sieving the samples were left to sediment (15–20 min) in a 250 ml conical beaker. After washing through the 45 µm sieve the sediment in the conical beaker was collected in a 15 ml centrifuge tube and centrifuged for 1 min at 100 *g*. The sediment was mixed with saline to form a 2.25 ml suspension. Small trematode eggs were counted in a total of 0.45 ml (dilution of the sample was often necessary) of each sample (100× magnification). This volume represented 1 g of faeces, and the counts were expressed as eggs per g of faeces (epg). In some cases less than five grams of faeces were obtained. The epg was then calculated as: Egg count×2.25(ml)/amount of faeces (g)×0.45(ml).

### Calculations and Statistical Analysis

The actual reinfection rates were calculated on day 30, 60, 90 and 120 as the cumulative number of dogs being copro-positive for FZT on the respective day divided by the number of dogs examined on the actual day. The Kaplan-Meier product limit method [20], commonly used in survival analysis to model the time to an event, was used to calculate the probability of being reinfected at a certain time point given that the dog stayed in the study up to that point. The model took into account information on dogs leaving the study before termination of the study due to various reasons and dogs still uninfected by termination of the study, through point censoring [20]. The median time till reinfection (the time when 50% of the dogs were expected to be reinfected) was estimated and difference between young (<1 year) and older dogs (≥1 year) was tested using the Kaplan-Meier model. The arithmetic mean of the intensities of infection among FZT-positive dogs on day 0 and 120 were compared within and between the treated group and likewise for the untreated group by Mann Whitney test (a non-parametric test had to be used since data was not normally distributed). Intensities of infection day 0 were also compared between young and older dogs using the same test as above. The effectiveness of the treatment was determined as number of treated dogs negative for undamaged eggs both on day 3 and 10 post treatment divided by the total number of treated dogs, multiplied by 100.

## Results

### Dog Keeping Practices

**Figure 1:**
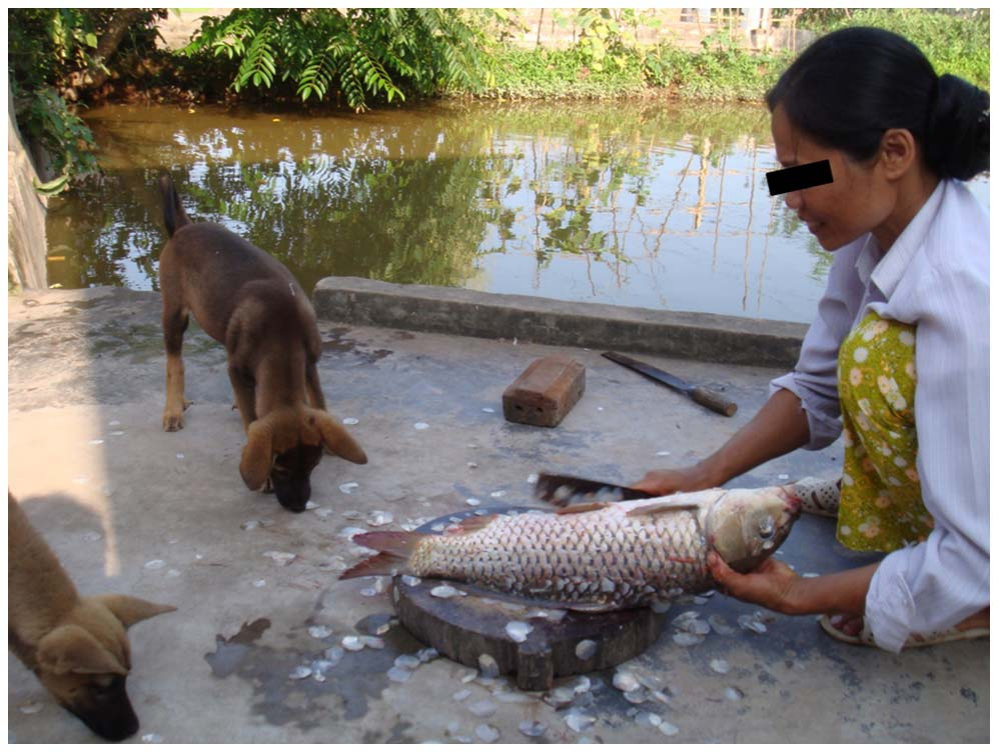
Processing of fish for dinner. A typical situation from Nghia Lac village in Nam Dinh Province, Vietnam, where a fish from the household's pond is prepared for dinner while free roaming dogs are allowed to eat scales and other fish leftovers from the preparation. Photo courtesy of Diana Sindberg.

A total of 73 farming households were visited for questionnaire interviews during the initial screening. The majority (86%) of the households had a fish pond in their close proximity (them self or neighbours). The households kept between 1 and 7 dogs, the majority having 1 or 2 dogs (38 and 36%, respectively). More than 80% of the households described that their dogs sometimes or always roamed freely. Forty-one percent reported that their dogs ate raw fish occasionally and 52% said the dogs ate intestines and other body parts when fish were prepared for household members. Other types of dog feed mentioned were rice, kitchen left overs and different types of cooked food. A typical situation with cleaning and preparation of fish for human consumption is illustrated in Fig. 1. Only 6 households had dewormed their dogs within the last month, 64% never dewormed their dogs, 21% dewormed the dogs once when they were young and a single farmer gave anthelmintic treatment twice per year. Most farmers did not remember which drug was used for treatment, though four farmers mentioned levamisol and a single farmer tetramisol as being the drug of choice. No drugs active against trematodes were mentioned. Twenty six female dogs and 18 males were included in the study (in three cases the sex was not recorded). The age of dogs <1 year ranged between 2–8 months (mean 2.4), dogs ≥1 year ranged between 1–7 years (mean 2.6). The body condition score (mean ± SD) was 2.4±0.5 meaning that majority of dogs were somewhat thinner than the ideal measure. All dogs were of mixed breed. This low number of older dogs in the treated group was due to some of the initially selected dogs being unavailable and selection of other dogs instead.

### Reinfection with FZT

**Figure 2:**
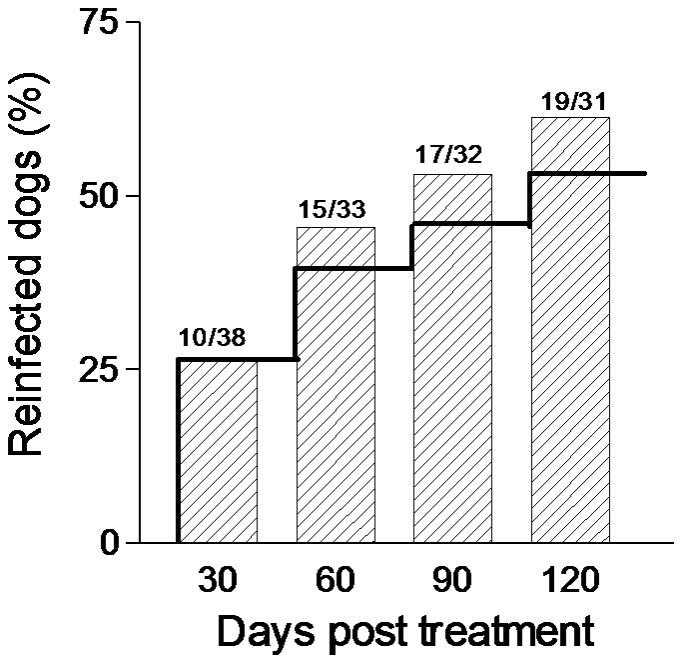
Reinfection rates with fish-borne zoonotic trematodes. The reinfection rates of dogs with fish-borne zoonotic trematodes after a single treatment with 40 mg/kg praziquantel. Bars: Actual reinfection proportions (cumulative number of dogs positive on the actual day divided by the number of dogs sampled on that particular day, calculation shown above columns). Curve: The Kaplan-Meier estimate for the reinfection rate.

**Figure 3:**
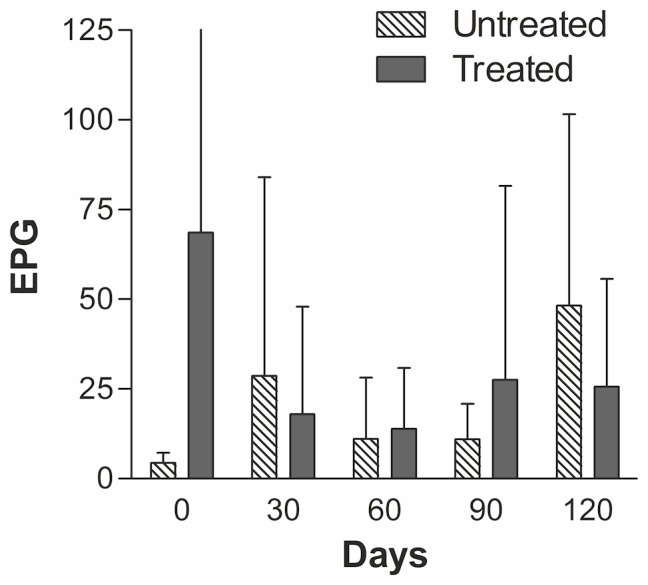
Intensities of small trematode eggs in dogs. Intensities of small trematode egg excretion in dogs (treated day 0 or untreated) expressed as mean number of eggs per g faeces (EPG) at different time points (group mean and standard deviation). The initial numbers of dogs were: untreated, N = 9 and treated N = 38.

The initial examination found 77/101 (76%) dogs positive for small trematode eggs in their faeces. Of the 38 dogs treated with praziquantel, 7 dogs dropped out prior to termination of the study, 12 remained uninfected and 19 became reinfected within the 120 days. The actual reinfection rates were 26.3% (day 30), 45.5% (day 60), 53.1% (day 90), 61.3% (day 120). This corresponded to 10 dogs becoming reinfected before day 30, additional 5 dogs between 30–60 days, 2 dogs between 60–90 and, finally, 2 dogs between 90–120 days post treatment. The reinfection rates and the Kaplan-Meier estimates can be seen in Fig. 2. The median time till reinfection was 105 days, (95% CI 60–120). The time till reinfection did not differ significantly between young and older dogs. Nineteen dogs were point censored in the study, of these, 7 dogs had left the study prior to the last sampling (day 30: 0, day 60: 5, day 90: 1, day 120: 1 dogs, respectively) because the dogs were sold or died. At the day of praziquantel treatment, the intensity of FZT infection in the dogs was 65±198 epg (mean ± SD) in the treated group and 4±3 epg in the untreated group, which differed significantly (P<0.05). No significant difference was seen in the intensity of infection between day 0 and 120 in any of the groups probably due to a large variation in egg counts. The fluctuations in intensity of infection and reinfection can be seen in Fig. 3. The effectiveness of the treatment was 100%. However, three days after treatment, few damaged eggs (1–4) with black spots or empty egg shells without operculum were found in three treated dogs and a similar findings was seen in three dogs at day 10 (one dog had such eggs both days). These damaged eggs were believed to be reminiscence of eggs trapped in the mucosa from the first infection and not a result of treatment failure. Two of these dogs showed, undamaged eggs at day 30 and one at day 60. The remaining two dogs dropped out of the study on day 60 and 120, respectively, without being reinfected. The untreated dogs remained positive throughout the study period.

## Discussion

The present study documented that reinfection of dogs with FZT took place within 30 days after praziquantel treatment. More than 25% of the dogs were reinfected day 30 and more than 50% at day 90 post treatment, respectively. The intensity of FZT infection four months after drug treatment was similar to the intensity found before treatment took place. Infected dogs kept excreting eggs for 4 months. In general, dogs were often exposed to raw fish; more than 50% through the feed and more than 80% roamed freely thereby having access to fish ponds. These practices are risk factors for infections [9], [10].

The initial prevalence found in this study showed that FZT infections are common in the dog population in this area as demonstrated in a study four years earlier (76% vs. 57% found in 2007) and the intensity of infection found initially in the treated dogs was higher than this earlier study [9]. Based on the Kaplan-Meier estimate more than 25% of treated dogs were reinfected one month after treatment and half of the treated dogs would be reinfected 105 days after treatment, a slightly more conservative estimate than the actual reinfection rates, due to the inclusion of censored dogs in the analysis. The results suggest that even if dogs were treated with praziquantel regularly, the likelihood of becoming reinfected soon after treatment would be very high, and thus continued contamination the environment with eggs would take place. The lack of apparent influence of the praziquantel treatment on the intensity of the reinfection is also important since it suggests that no immunity, or immunity of limited strength, is acquired following treatment. Twelve dogs (32%) remained uninfected at day 120 perhaps due to lack of, or infrequent, exposure to raw fish. The present findings are also alarming from a human treatment perspective: If reinfections occur within months after treatment, obviously more frequent treatments would be needed to control the FZT. This again would lead to higher risk for development of resistance against praziquantel. Monitoring mass drug treatment programs in humans, which primarily aim at targeting the liver flukes, are further complicated by the presence of MIF egg which cannot be distinguished from the liver fluke eggs [6]. Hence proper diagnostic tools to differentiate between liver fluke and MIF eggs are urgently needed.

For dogs, several factors favour reinfection: Fish ponds are common in the villages and most dogs roam freely with access to ponds and fish. A previous study in the same area found that the main risk factor for having infection with FZT was if the dogs were fed raw fish [9]. In the present study more than 50% of the households gave their dogs access to raw fish left over.

Most of the dog in the current study was less than one year old. This is a result of the farm practice in the area, where many dogs are kept for meat, hence are sold for consumption at around one year of age. Despite the skewed age profile, no significant difference was found in reinfection rate or intensity of infection comparing dogs younger and older than one year.

When calculating the effectiveness a criterion of negativity for intact eggs on day 3 and 10 post infection was used. Hence, the 5 dogs excreting damaged eggs were regarded cured of the FZT infection and the effectiveness of the treatment in the field to be 100%. In a previous study in dogs and cats where two different doses of praziquantel were evaluated, two cats receiving 75 mg/kg also showed damaged eggs 3 days post treatments that were judged non-viable [15]. We therefore reason that our assumption is valid. The finding of damaged eggs could be due to worms or eggs being trapped in the mucosa and therefore being expelled over some days. Excretion of non-viable eggs for days after treatment is known to occur e.g. with *Schistosoma japonicum* [21], [22]. Earlier studies have assessed the cure rate of a single praziquantel dose of 40 mg/kg against opisthorchiasis in humans and have found it to be 91 and 95.5% [23], [24]. No strict rules apply for defining effectiveness and based on faecal examinations alone, no true cure rate can be obtained. Future studies should evaluate the true cure rate of praziquantel on MIF by doing necropsies and worm recovery after treatment.

A recent intervention study for reducing prevalence and intensity of fish-borne zoonotic trematode infections in The Red River Delta in Vietnam, confirmed the importance of including dogs and cats in the control programs [25]. However, the authors also pointed out, that the practicality of control programs must be considered, and in this regard, treatment of domestic animals and humans is costly and difficult to undertake. Combined with the knowledge gained in the present study about the high reinfection rate and the existing treatment practise of dogs for helminths, recommending farmers to treat their dogs for trematodes with frequent intervals is not a sustainable way to overcome infections with FZT. Awareness about the need for an integrated approach towards control of FZT is rising e.g. as described by Sithithaworn et al. [26] who suggest a community-orientated approach including treatment, improved sanitation and information, education and communication. Our recommendation is to include teaching about feeding practise of the dogs and other domestic animals in such a program to avoid reoccurring reinfections to take place.

To conclude, dogs have easy access to raw fish and do not receive treatment against flukes by their owner. After a single treatment more than 50% of the dogs were reinfected after 90 days. The effectiveness of the single dose of praziquantel was found to be 100%. Treatment of individual dogs with symptoms of FZT infection is of course always recommendable, however, repeated mass treatments are hardly applicable and cannot stand alone for control of FZT in dogs.
